# Investigating the Impact of Surfactant-Based Warm-Mix Additives on the Performance of Recycled Asphalt Mixtures

**DOI:** 10.3390/ma18081732

**Published:** 2025-04-10

**Authors:** Hao Xiang, Desheng Yang, Shunxian Peng, Wei Gao

**Affiliations:** 1 College of Civil Engineering and Architecture, Southwest University of Science and Technology, Mianyang 621010, China; 2 National & Local Joint Engineering Research Center of Transportation and Civil Engineering Materials, Chongqing Jiaotong University, Chongqing 400074, China; 3 Shuicheng Highway Administration Bureau, Liupanshui 553001, China; 4 Guizhou Industry Polytechnic College, Guiyang 551400, China

**Keywords:** recycled asphalt mixtures, warm-mix additives, performance evaluation, rheological properties, water stability

## Abstract

This investigation aimed to assess the influence of warm-mix additives on the performance characteristics of recycled asphalt mixtures. Pressure-aging vessels were employed to simulate the aging of asphalt binders. Warm-mix recycled asphalt (WMRA) and mixtures were prepared by incorporating self-developed plant-oil-based rejuvenators and surfactant-based warm-mix additives. The rheological properties of asphalt were tested by a dynamic shear rheometer (DSR). Furthermore, the pavement performance of the asphalt mixture was evaluated by a rutting test, beam bending test, Marshall stability test, and freeze–thaw splitting test. The experimental results demonstrated that the addition of warm-mix additives reduces the penetration and softening point of recycled asphalt while enhancing its ductility. Performance improvement was quantitatively evaluated using a recovery index. The complex modulus and rutting factor of the WMRA were found to be lower than those of recycled asphalt, indicating a decrease in the asphalt’s resistance to deformation owing to the surfactant. Both the hot-mix and warm-mix recycled asphalt mixtures met the specified requirements for various performance indicators. The warm-mix rejuvenator outperformed the regular rejuvenator in evaluating water stability using the soaked Marshall residual stability method, whereas the evaluation based on the freeze–thaw splitting strength ratio demonstrated the opposite trend.

## 1. Introduction

Asphalt pavement recycling technology represents a significant advancement in sustainable road construction practices [1]. Recycling technology for reclaimed asphalt pavement (RAP) refers to the recycling of waste materials generated during the process of asphalt pavement rehabilitation, as well as during the production and paving of asphalt mixtures. After crushing and screening, RAP is mixed with recycling agents, new aggregates, and new asphalt to produce recycled asphalt mixtures that meet the performance requirements for road applications [2]. Warm-mix asphalt technology [3] is characterized by reducing the mixing and compaction temperatures. By combining recycling technology with warm-mix technology, it is possible to increase the RAP blending ratio and reduce the heating temperature requirements [4]. The favorable social and economic benefits of warm-mix recycling technology have gained increasing attention from scholars.

Yousefi [5] conducted a research study focused on evaluating the performance of warm-mix recycled asphalt (WMRA) mixtures for road applications and revealed the importance of additive types on pavement mechanical properties. Wang [6] evaluated the suitability of WMRA mixtures based on their fatigue and low-temperature performance. The study concluded that warm-mix technology can be used for the recycling of asphalt with a 40% RAP content. Caputo [7] provided an overview of the mechanisms of popular warm-mix additives and the warm-mix asphalt (WMA) mixing process, and the environmental and technological advantages of the warm mix compared to traditional hot-mix asphalt were also introduced in the aforementioned study. Chen [8] affirmed the role of WMA in reducing viscosity and promoting environmental sustainability through the study of the effect of warm-mix technology on the performance of aged asphalt. Gong [9] studied the influence of warm-mix additives on asphalt viscosity, glass transition temperature (Tg), damping properties, mechanical performance, and phase separation morphology. Hettiarachchi [10] provided a review of previous research, which indicated that the application of warm-mix technology in RAP recycling can improve the resistance to rutting, enhance mixing uniformity, and have a positive effect on the low-temperature performance of asphalt mixtures; however, conducting further investigations to improve the accuracy of the results was suggested.

Although numerous achievements have been made in the fields of asphalt recycling and warm-mix technology, research regarding warm-mix recycled asphalt mixtures remains in the exploratory stage. Scholars from both domestic and international institutions have primarily focused on specific warm-mix additives; further research is needed to diversify these studies. In this study, starting from raw materials and utilizing surface activity technology, the influence of warm-mix additives and rejuvenators on the physical and rheological properties of aged asphalt was analyzed. Furthermore, considering the use of RAP materials in engineering applications, the performance of WMRA mixtures was evaluated, and the effects of different recycling methods were compared.

## 2. Materials and Methods

### 2.1. Raw Materials

#### 2.1.1. Asphalt

Sinopec DH70# was used as the original asphalt. To simulate the long-term aging of asphalt, the original asphalt was subjected to aging experiments using a rotating thin-film oven test (163 °C for 85 min) and a pressure-aging vessel test (2.1 MPa at 110 °C for 48 h), resulting in the aged asphalt denoted as DH70#PAV. Table 1 presents the basic parameters of the original and aged asphalts. A mixture asphalt (MA) was prepared by blending original and aged asphalt at a 2:3 ratio, simulating a high-RAP-content (30~50%) scenario in recycled asphalt production.

#### 2.1.2. Rejuvenator

The rejuvenator used in this study was a plant-oil-based one, mainly composed of linseed oil [11]. Table 2 shows its basic parameters. Adding 9% of this rejuvenator to the asphalt mixture created rejuvenated asphalt, denoted as RA.

#### 2.1.3. Warm-Mix Additive

The warm-mix additive used in this study was composed of three surfactant components. Component A is a monoglyceride of stearic acid, which has no irritating odor and is soluble in oils, alcohols, and other organic solvents. It has a melting point of 65 °C and a hydrophilic–lipophilic balance value of less than 6. It functions as an emulsifier, dispersant, wetting agent, and viscosity regulator. Component B is oleic diethanolamide, which is soluble in water, propylene glycol, methanol, and other organic solvents. It has an HLB value of 4.3 and exhibits properties such as emulsification, plasticization, lubrication, and dispersion. Component C is S80, which has no irritating odor and is easily soluble in water. It exhibits excellent foaming, penetration, lubrication, and emulsifying properties. It is often used as a viscosity regulator with a carbon atom count of 11 and an HLB value of 7.5. The proportions of components A, B, and C are given by the ratio of 12:7:6. During the preparation of the warm-mix additive, component A was heated until completely melted, at a temperature of 50–60 °C. Subsequently, components B and C were added in proportion, followed by thorough stirring at a constant temperature. The warm-mix additive and rejuvenator were blended in a 7:3 ratio to formulate the warm-mix rejuvenator. Incorporating 9% of this rejuvenator into the asphalt mixture yielded WMRA.

#### 2.1.4. Mixture Proportion Design

**Figure 1:**
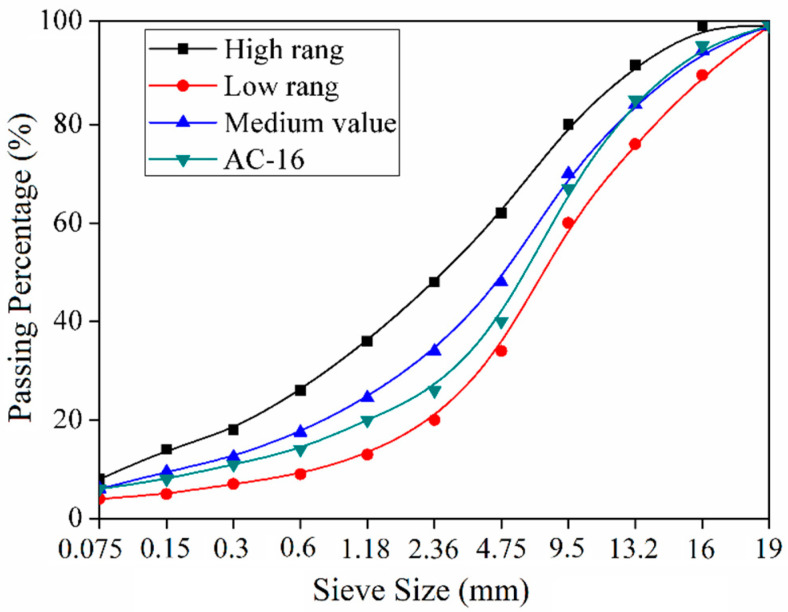
Gradation of recycled asphalt mixtures.

This study utilized RAP obtained from the G60 expressway in Guizhou, China, with a reclaimed material content of 40%. The new aggregates consist of two gradations of limestone: 0–10 mm and 10–20 mm. Limestone mineral powder was selected as the mineral filler. The designed mixture was an AC-16 type recycled asphalt mixture with 4.9% asphalt content. Figure 1 shows the mixture grading curve.

### 2.2. Experimental Methods

#### 2.2.1. Physical Performance Tests

In order to accurately reflect the influence of the warm-mix rejuvenator on the performance of mixed asphalt, conventional performance indicators of asphalt samples were tested. This study proposes the performance recovery index as a quantitative evaluation index. As shown in Formula (1), each index can be used to evaluate the degree of influence of the warm-mix rejuvenator on the performance of the mixed asphalt. A value of 0 indicates no effect, whereas 1 indicates that the indicator returns to the original asphalt state.

(1) $$\left\{\begin{array}{l} PI=\left|\frac{PI_{i}-PI_{mix}}{PI_{original}-PI_{mix}}\right|\\ RH=\left|\frac{RH_{i}-RH_{mix}}{RH_{original}-RH_{mix}}\right|\\ YD=\left|\frac{YD_{i}-YD_{mix}}{YD_{original}-YD_{mix}}\right|\\ ND=\left|\frac{ND_{i}-ND_{mix}}{ND_{original}-ND_{mix}}\right| \end{array}\right.$$

Here, PI, RH, YD, and ND represent the recovery index of penetration, softening point, ductility, and viscosity, respectively. The subscript i indicates the asphalt performance parameters when the additive content is i%.

#### 2.2.2. Dynamic Shear Rheological Experiment

The complex modulus ($G^{*}$) and phase angle (δ) of the asphalt were tested using a dynamic shear rheometer (DSR). By analyzing the changes in the viscoelastic properties and anti-rutting factor of asphalt, the effect of the regeneration method on the rheological properties of asphalt was explored [12]. The sample thickness was 1 mm, and the diameter was 25 mm. The temperature scan mode was selected during the test; the temperature ranged between 40 and 64 °C with an interval of 6 °C. The stress control value was 0.12 Pa, and the frequency was 10 Hz.

#### 2.2.3. High-Temperature Rutting Experiment

Dynamic stability can effectively reflect the ability of asphalt pavements to resist permanent deformation under high-temperature conditions [13,14]. In this study, the dynamic stability of the asphalt mixture was tested using a wheel-tracking test. The wheel tracking specimens were formed with a wheel pressure of 0.7 MPa and had the following dimensions: 300 mm in length, 300 mm in width, and 50 mm in thickness. The dynamic stability test was conducted with a rolling speed of 42 cycles per minute at a temperature of 60 °C for a rolling duration of 60 min.

#### 2.2.4. Low-Temperature Fracture Resistance Experiment

Recycled asphalt pavement is susceptible to the effects of temperature shrinkage stress at lower temperatures [15]. This study employed the low-temperature small-beam bending test method to evaluate the low-temperature fracture resistance of recycled asphalt mixtures. Test specimens were prepared using the aforementioned procedure and cut into prism-shaped small-beam specimens with dimensions of 250 mm in length, 30 mm in width, and 35 mm in height. The test was conducted with a span of 200 mm and at a temperature of −10 °C. By measuring the maximum load and mid-span deflection at the point of specimen failure, the maximum tensile strain (Equation (2)), flexural tensile strength (Equation (3)), and flexural stiffness modulus (Equation (4)) were calculated:

(2) $$\varepsilon_{B}=\frac{6\times h\times d}{L^{2}}$$

(3) $$R_{B}=\frac{3\times L\times P_{B}}{2\times b\times h^{2}}$$

(4) $$S_{B}=\frac{R_{B}}{\varepsilon_{B}}$$

Here, $\varepsilon_{B}$ is the maximum tensile strain (με) at the point of specimen failure, $R_{B}$ is the flexural tensile strength (MPa), $S_{B}$ is the flexural stiffness modulus (MPa) at the point of specimen failure, and b, h, and L are the width (mm), height (mm), and span (mm) of the cross-section of the specimen, respectively.

#### 2.2.5. Water Stability Test

Water damage is a typical type of early distress for asphalt pavements [16,17]. In this study, two indicators, namely the soaked Marshall residual stability and freeze–thaw splitting strength ratio, were used to comprehensively evaluate the water stability of asphalt mixtures. In the soaked Marshall test, two sets of Marshall specimens were separately conditioned for 30 min and 48 h at 60 °C. The residual stability of the specimens was calculated using Equation (5). In the freeze–thaw splitting test, the first set of specimens was immersed in a water bath at 25 °C for 2 h, and the experimental load value was recorded. The second set of specimens underwent vacuum saturation at 98 kPa, followed by freezing at −18 °C for 16 h, which were then conditioned in water at 60 °C for 24 h. The experimental load value was recorded, and the freeze–thaw splitting strength ratio (TSR) was calculated using Equation (6).

(5) $$MS=\frac{MS_{1}}{MS_{0}}\times 100\%$$

(6) $$TSR=\frac{R_{T2}}{R_{T1}}\times 100\%$$

Here, MS is the Marshall residual stability of the asphalt mixture (%), $MS_{1}$ represents the Marshall stability after 48 h of soaking (kN), and $MS_{0}$ is the Marshall stability after 30 min of soaking (kN).

## 3. Analysis of Experimental Results

### 3.1. Physical Index Results

**Figure 2:**
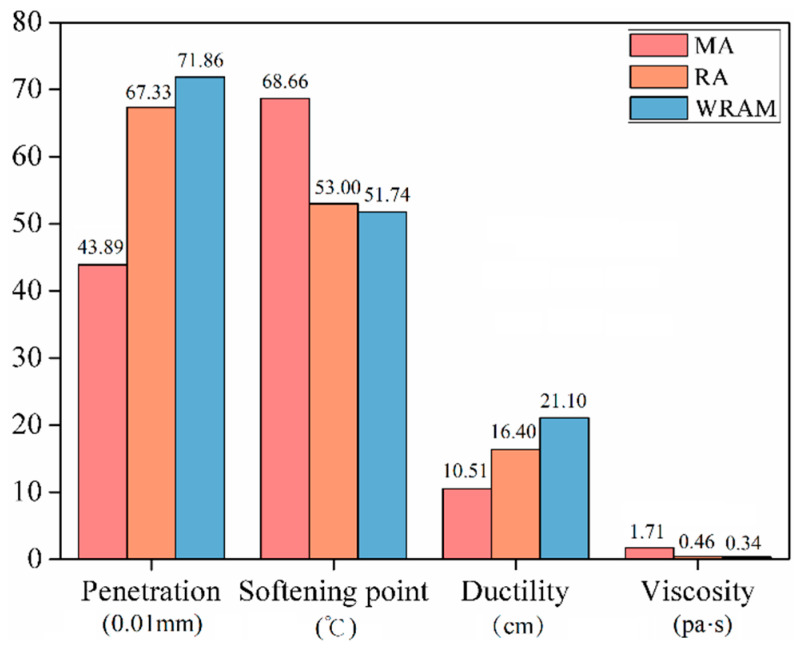
Physical properties of asphalt.

**Figure 3:**
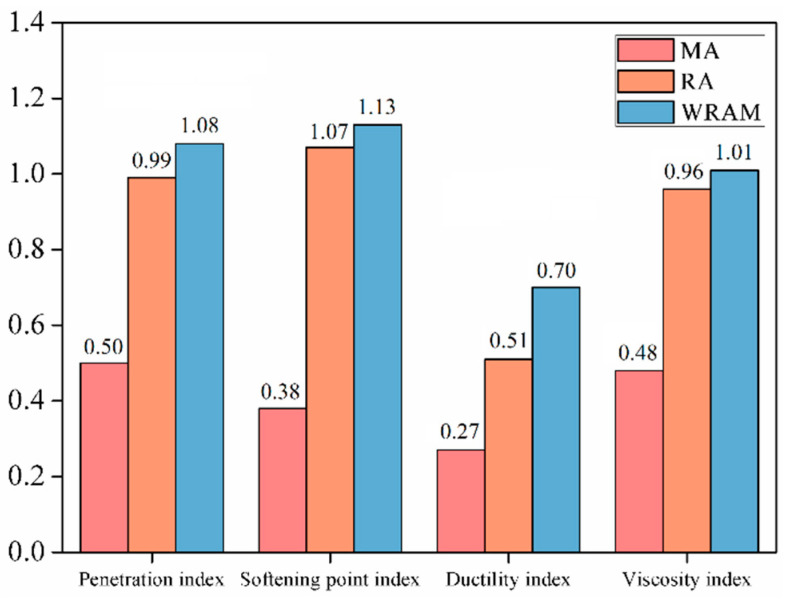
Recovery index of asphalt physical properties.

Figure 2 and Figure 3 illustrate the physical parameters and recovery indices of the asphalt mix and regenerated asphalt. After regeneration, the penetration and softening points of the asphalt mix increased, whereas the softening point and viscosity decreased. The penetration recovery indices for the asphalt mix, regenerated asphalt, and warm-mixed regenerated asphalt were 0.5, 0.99, and 1.08, respectively. These results indicate that both the regenerant and warm-mixed regenerant can replenish the chemical components in aged asphalt, leading to improved penetration. The warm-mixed regenerant exhibits a better recovery effect on asphalt penetration compared to the regenerant.

The softening point recovery index of the asphalt mix was 0.38, which was slightly reduced compared to that of the aged asphalt, but the difference was not significant. This can be attributed to the high degree of aging of the asphalt and the high content of aged asphalt in the mix. The softening point indices for the regenerated asphalt and warm-mixed regenerated asphalt were 1.07 and 1.13, respectively. Both the regenerant and warm-mixed regenerant can effectively restore the softening point of the asphalt. The warm-mixed regenerant exhibited stronger regeneration effects than those of the regenerant.

The ductility of the aged asphalt demonstrated an increasing trend with the addition of base asphalt, regenerant, and warm-mixed regenerant. The ductility recovery indices for the asphalt mix, regenerated asphalt, and warm-mixed regenerated asphalt were 0.27, 0.51, and 0.7, respectively. None of the regeneration methods in this study could restore the ductility of the aged asphalt to the level of the base asphalt. However, the warm-mixed regenerated asphalt specimens exhibited a more advantageous ductility recovery. This is mainly because the surface-active materials improve the polarity of aged asphalt, aligning it closer to the polarity of the base asphalt and enhancing the compatibility between the aged asphalt, base asphalt, and regenerant [18,19].

The addition of the regenerant and warm-mixed regenerant to the asphalt mix effectively restores the viscosity. The viscosity indices for the asphalt mix, regenerated asphalt, and warm-mixed regenerated asphalt at 135 °C were 0.48, 0.96, and 1.01, respectively. The warm-mixed regenerant exhibited a better viscosity recovery effect on the asphalt. This is mainly because the surface-active components in the warm-mixed regenerant improve the polarity of the asphalt mix, thereby enhancing the ability of the asphalt to dissolve the regenerant.

### 3.2. Dynamic Shear Rheological Test Results

Dynamic shear rheological testing is an important method for evaluating the rheological properties of asphalts. The complex shear modulus (G*) and phase angle (δ) are crucial parameters that reflect the viscoelastic behavior of asphalt. The Strategic Highway Research Program (SHRP) in the United States defines G*/sinδ as the rutting factor, which is used to characterize the ability of asphalt binders to resist permanent deformation under high-temperature conditions. This parameter is significant for assessing the asphalt binder resistance to permanent deformation under high-temperature conditions.

**Figure 4:**
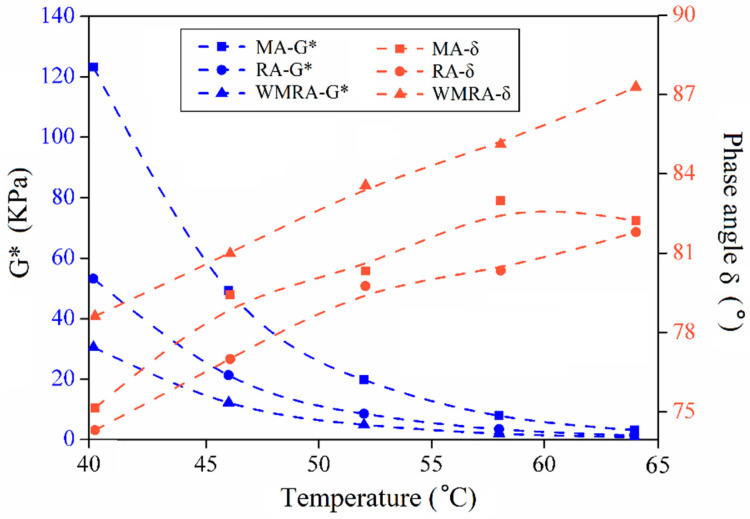
Relationship between temperature and G*, δ.

**Figure 5:**
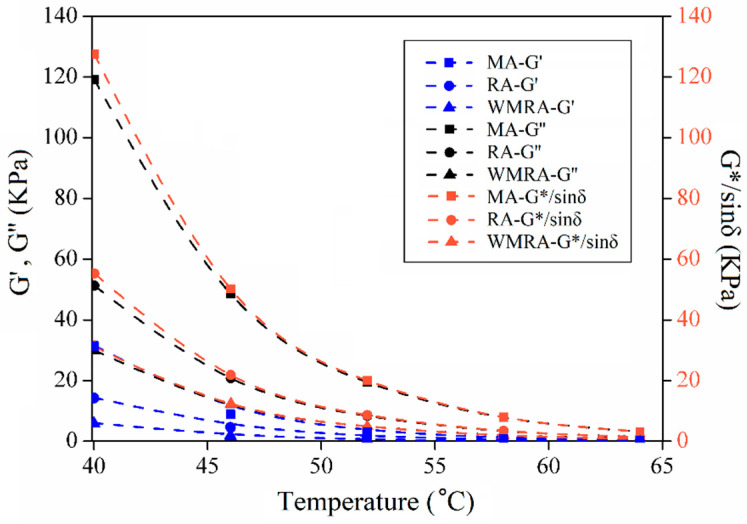
Relationship between temperature and G′, G″, and G*/sinδ.

Figure 4 and Figure 5 illustrate the relationship between the temperature and rheological parameters. The variations in these parameters in different asphalt samples exhibit a consistent trend. As the temperature increases, δ tends to increase, while G* and G*/sinδ tend to decrease. These trends indicate an increasing proportion of viscous components and a decreasing proportion of elastic components in the asphalt, leading to a reduced ability to resist deformation. The complex moduli of the asphalt samples, from highest to lowest, were observed in the following order: MA, RA, and WMRA.

As the temperature increases, the proportion of G′ decreases, while the proportion of G″ increases in the asphalt samples. During the shearing process, the recoverable deformation decreases, while the irrecoverable deformation increases. MA exhibits higher viscous and elastic moduli than RA, and the decreasing trend of the elastic modulus with temperature is less pronounced in MA, whereas the decreasing trend of the viscous modulus is more significant. This indicates that the mechanical properties of aged asphalt gradually transition from viscoelastic to elastic [20].

When comparing the rutting resistance factors of several asphalt types, MA exhibited the highest rutting resistance factor at the same temperature. This indicates an enhanced ability to resist high-temperature deformation after asphalt aging. However, the addition of rejuvenators and warm-mix additives resulted in a decrease in the rutting resistance factor of asphalt, suggesting an adverse effect of rejuvenators on the high-temperature performance of aged asphalt. Specifically, WMRA demonstrates a lower rutting resistance factor compared to RA, indicating that surface-active agents reduced the resistance of asphalt to deformation [21,22].

### 3.3. High-Temperature Rutting Test Results

The high-temperature stability of asphalt mixtures refers to the ability of asphalt pavements to resist significant plastic deformation under repeated vehicle loading during the summer or elevated temperatures. In the Chinese specifications, the dynamic stability index is adopted to effectively reflect the ability of asphalt pavements to resist permanent deformation under high-temperature conditions. The experimental results of this study are presented below.

**Figure 6:**
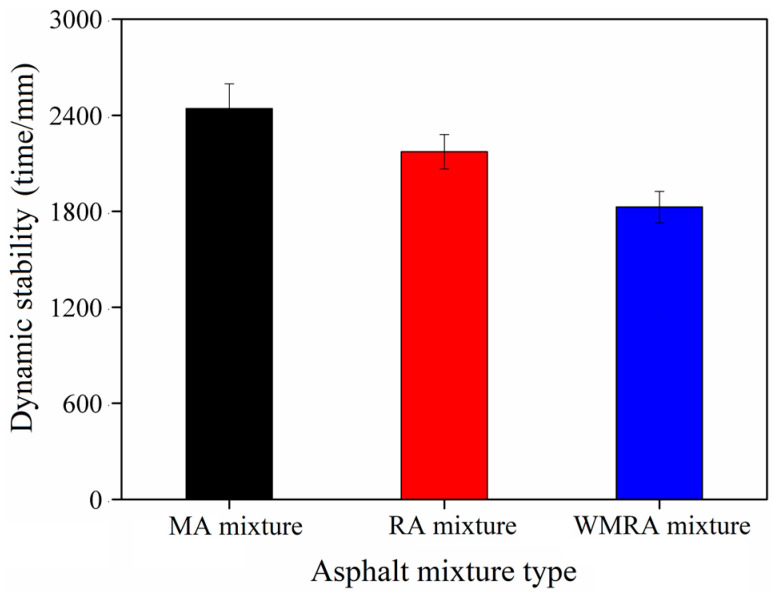
Dynamic stability of asphalt mixtures.

Figure 6 demonstrates that the dynamic stability values of the asphalt mixtures without the rejuvenator, with the rejuvenator, and while using a warm-mix rejuvenator were 2441, 2172, and 1826 cycles/mm, respectively. All the values meet the requirement specified in the regulations, which stipulates a minimum of 800 cycles/mm. These results are consistent with the findings of the dynamic shear rheological tests on reclaimed asphalt, indicating an improvement in the deformation resistance following asphalt aging. This improvement can be attributed to the increased proportion of aged asphalt in the reclaimed asphalt mixture, which results in a higher asphalt viscosity and enhanced stiffness of the mixture. Consequently, the high-temperature performance of the mixture was enhanced, leading to improved resistance to deformation.

### 3.4. Low-Temperature Performance Test Results

Low-temperature crack resistance refers to the ability of asphalt pavements to withstand the formation of low-temperature shrinkage cracks when subjected to cold temperatures. Given the presence of aged asphalt with a higher viscosity in reclaimed asphalt mixtures, studying the low-temperature performance of these mixtures is crucial [23]. In this study, the evaluation of the low-temperature performance of asphalt mixtures primarily employed the beam bending test, where the ultimate flexural strain served as the primary evaluation parameter for the low-temperature performance of the beam.

**Figure 7:**
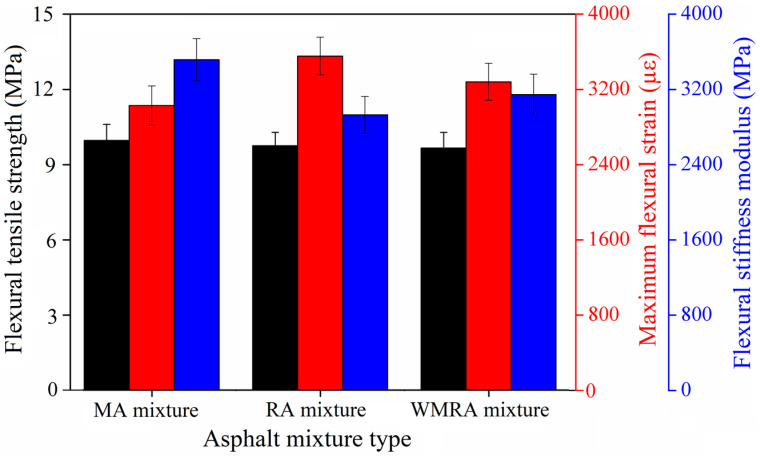
Low-temperature performance test results of asphalt mixtures.

Figure 7 presents the test results of the low-temperature crack resistance experiments conducted on the asphalt mixtures. All three methods used to obtain the asphalt mixtures met the requirement specified in the regulations, which states that the flexural strain should not be less than 2000 με. The differences in the failure flexural strengths of the specimens were minimal. Among the three methods, the RA mixture exhibited the highest flexural failure strain, followed by the WMRA and MA mixtures. This indicates that both the rejuvenator and warm-mix rejuvenator have a positive effect on improving the low-temperature performance of the asphalt mixtures. Furthermore, the rejuvenator demonstrated a greater improvement in low-temperature performance compared to the warm-mix rejuvenator.

The failure flexural modulus of elasticity of the asphalt mixture decreased in the following order: without the rejuvenator, with the warm-mix rejuvenator, and with the rejuvenator. Both the rejuvenator and warm-mix rejuvenator reduced the flexural modulus of elasticity of the asphalt mixture. This reduction can be attributed to the softening effect of the rejuvenator on aged asphalt, which enhances the stress relaxation capacity of aged asphalt under low-temperature conditions.

### 3.5. Water Stability Test Results

The water stability of asphalt pavements refers to their ability to resist damage caused by water infiltration [24]. Water damage to asphalt pavements occurs owing to the combined effects of water and vehicle loads. Water primarily acts on the pores and cracks within the asphalt pavement, exerting dynamic hydraulic pressure under the influence of the vehicle tire load. This leads to the continuous diffusion of water molecules into the interface between the asphalt and aggregates, resulting in a reduction in the adhesion between the asphalt and aggregates. Consequently, the integrity of the asphalt mixture is compromised, leading to a decrease in its ability to undergo ductile deformation.

In this study, the Marshall residual stabilities of the asphalt mixture before and after immersion in water and the residual tensile strength ratio after freeze–thaw cycling were measured separately to evaluate water stability.

**Figure 8:**
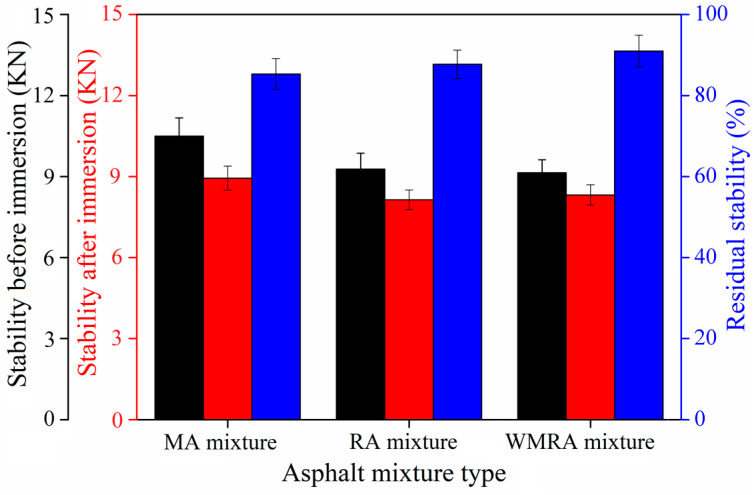
Residual stability of asphalt mixtures.

**Figure 9:**
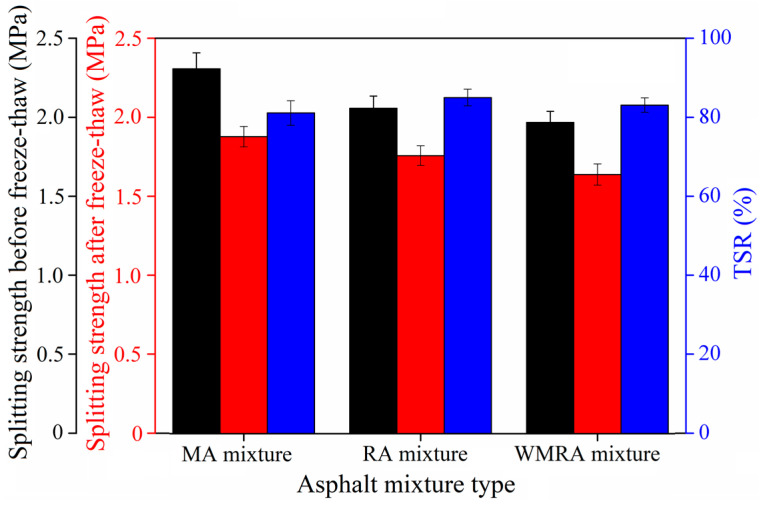
TSR of asphalt mixtures.

Figure 8 and Figure 9 depict the Marshall residual stability and residual splitting strength ratio of the asphalt mixtures, respectively. All three methods used to obtain the asphalt mixtures exhibit a water stability performance that meets the requirements for practical use. After immersion in water and undergoing freeze–thaw cycling, the Marshall stability and splitting tensile strength of the asphalt mixtures decreased.

When evaluating the water stability of the mixtures using the immersion Marshall residual stability test, the water stability of the mixtures obtained using the three rejuvenation methods ranked from high to low as follows: WMRA > RA > MA. When they were evaluated using the freeze–thaw splitting test, the ranking was RA > WMRA > MA. These results indicate that both the rejuvenator and warm-mix rejuvenator improve the water stability of the asphalt mixtures.

## 4. Conclusions

In this study, laboratory tests were conducted to evaluate the physical and rheological properties of warm-mix recycled asphalt by selecting suitable warm-mix additives and rejuvenators. Furthermore, the performance of the warm-mix asphalt mixture was assessed through wheel tracking, small-beam low-temperature bending, and freeze–thaw splitting tests. The summarized experimental results are as follows:

(1) Compared to base asphalt, aged asphalt exhibits a decrease in penetration and ductility, as well as an increase in the softening point. With the addition of the base asphalt, rejuvenator, and warm-mix rejuvenator, the penetration decreases, the softening point decreases, and ductility increases. Both rejuvenation methods were found to restore the penetration and softening points of the aged asphalt to those of the base asphalt. However, the restorative effect on the ductility of asphalt was not satisfactory for either of the rejuvenation methods.
(2) The addition of fresh asphalt, rejuvenator, and warm-mix rejuvenator to aged asphalt can effectively reduce its viscosity. The restorative effect on the workability performance of the asphalt varied; the warm-mix rejuvenator demonstrated the highest effect, followed by the rejuvenator and base asphalt. Both the warm-mix rejuvenator and rejuvenator decrease the resistance of asphalt to deformation under high-temperature conditions.
(3) The performance indicators of the recycled asphalt mixture met the specifications. The warm-mix rejuvenator and rejuvenator reduced the high-temperature stability of the mixture while enhancing its low-temperature and water stability. This is primarily owing to the softening effect of the rejuvenators on the asphalt, which leads to a decrease in its deformation resistance. Additionally, the rejuvenators improved the stress relaxation capability of asphalt under low-temperature conditions.
(4) Warm-mix rejuvenation technology, which achieves energy conservation and emission reduction by lowering the temperature, is a green and environmentally friendly pavement construction technique developed in response to the current global challenges of energy depletion and severe atmospheric pollution. In the future, the focus of research should be on the development of more high-performance and cost-effective warm-mix additives.
(5) Future research should concentrate on the impacts of diverse climate conditions on the performance of warm-mix recycled asphalt mixtures. It is crucial to optimize the mix design for enhanced environmental adaptability and explore eco-friendlier additive preparation methods to further boost the sustainable development potential of warm-mix recycling technology.

## Figures and Tables

**Table 1:** Basic parameters of asphalt.

Type	Penetration at 25 °C/0.1 mm	Softening Point/°C	Ductility at 15 °C/cm	Viscosity at 135 °C
DH70#	67.9	54.6	>150	0.356
DH70#PAV	19.9	77.4	7	2.940

**Table 2:** Rejuvenator technical parameters.

Viscosity at 135 °C/Pa·s	Mass Loss After RTFOT/%	Viscosity Ratio After RTFOT	Flash Point/°C	Relative Density
0.0072	2.74	0.77	260	0.931

## Data Availability

The original contributions presented in this study are included in the article. Further inquiries can be directed to the corresponding author.
